# Supplementary material for: Larp1 supports brain growth and spatial memory via post-transcriptional control of the translation machinery
Source: bioRxiv. 2025 Oct 10:2025.10.09.681478. Preprint. [Version 1] doi: 10.1101/2025.10.09.681478 (PMC12632453; doi:10.1101/2025.10.09.681478)
Supplement: Supplement 1 [file NIHPP2025.10.09.681478v1-supplement-1.pdf]

bioRxiv preprint doi: https://doi.org/10.1101/2025.10.09.681478; this version posted October 10, 2025. The copyright holder for this preprint (which was not certified by peer review) is the author/funder, who has granted bioRxiv a license to display the preprint in perpetuity. It is made available under aCC-BY 4.0 International license.

## Supplemental Information

bioRxiv preprint doi: https://doi.org/10.1101/2025.10.09.681478; this version posted October 10, 2025. The copyright holder for this preprint (which was not certified by peer review) is the author/funder, who has granted bioRxiv a license to display the preprint in perpetuity. It is made available under aCC-BY 4.0 International license.

**Supplemental Table 1.**

Differential gene expression between Larp1 WT and cKO cortex.

**Supplemental Table 2.**

TOPscores for genes in mouse cortex.

bioRxiv preprint doi: https://doi.org/10.1101/2025.10.09.681478; this version posted October 10, 2025. The copyright holder for this preprint (which was not certified by peer review) is the author/funder, who has granted bioRxiv a license to display the preprint in perpetuity. It is made available under aCC-BY 4.0 International license.

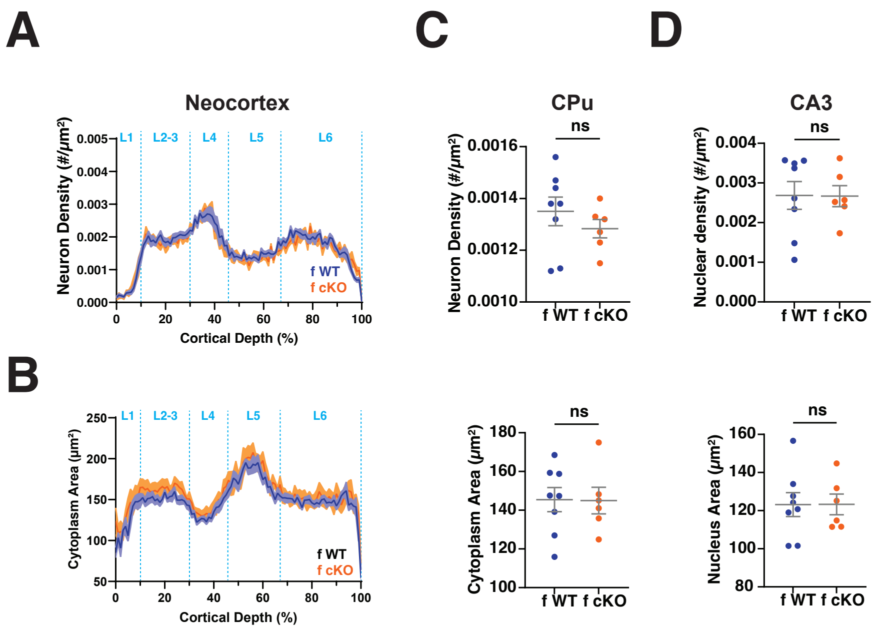


**Supplemental Fig 1. Neuron size and density in female mice. A.** The density of cortical neurons in Larp1 WT and cKO female mice. NeuN+ cells from regions of the primary somatosensory region from female Larp1 WT and cKO mice were segmented and binned by position along the vertical axis in 1% increments. Plot shows mean number and SEM within each bin. Significance by REML group, n=8 WT and 6 cKO. **B.** Sizes of neuronal soma in female Larp1 WT and cKO mice. Areas of NeuN+ cells in segmented regions from (A) for female Larp1 WT and cKO mice. Traces are means +/- SEM. Significance by REML group p value = 0.0257, n=8 WT and 6 cKO. **C.** Neuronal density in the caudate putamen of female Larp1 WT and cKO mice. NeuN+ cells in the caudate putamen (CPu) of female Larp1 WT and cKO mice were segmented and analyzed as in (A). Top panel: density of NeuN+ cells from the indicated genotypes. Significance by t-test, n n=8 WT and 6 cKO. Bottom panel: area of NeuN+ cells from the indicated genotypes. Significance by t-test, n=8 WT and 6 cKO. **D.** Density of nuclei in the CA3 region of the hippocampus of female Larp1 WT and cKO mice. DAPI+ nuclei from the CA3 region were segmented and analyzed as in (A). Top panel: density of DAPI+ nuclei in the indicated genotypes. Significance by t-test, n=8 WT and 6 cKO. Bottom panel: area of DAPI+ nuclei from the indicated genotypes. Significance by t-test, n=8 WT and 6 cKO.

bioRxiv preprint doi: https://doi.org/10.1101/2025.10.09.681478; this version posted October 10, 2025. The copyright holder for this preprint (which was not certified by peer review) is the author/funder, who has granted bioRxiv a license to display the preprint in perpetuity. It is made available under aCC-BY 4.0 International license.

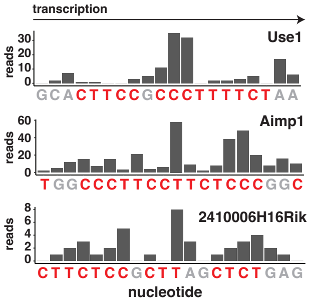


**Supplemental Fig 2. Transcription start sites of non-canonical mRNAs.** Examples of TOP motifs in mRNAs depleted from Larp1 cKO mice. Transcription start sites for the indicated genes from FANTOM5 CAGE data analysis of mouse neonatal cortex (see Methods).

bioRxiv preprint doi: https://doi.org/10.1101/2025.10.09.681478; this version posted October 10, 2025. The copyright holder for this preprint (which was not certified by peer review) is the author/funder, who has granted bioRxiv a license to display the preprint in perpetuity. It is made available under aCC-BY 4.0 International license.

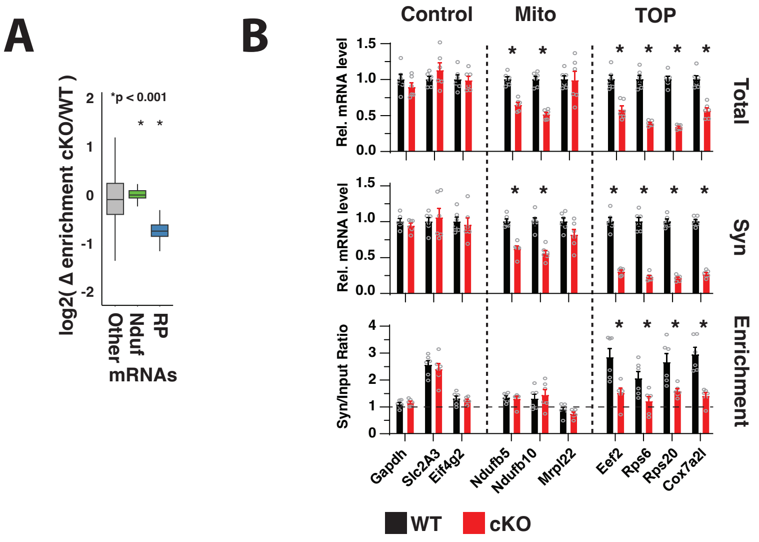


**Supplemental Fig 3. Synaptosome enrichment of TOP and non-TOP mRNAs in synaptosomes from WT and Larp1 cKO brains. A.** Complex I (Nduf) mRNAs are not depleted from synaptosomes in Larp1 cKO brains. Plot shows changes in the synaptosome enrichment (synaptosome/total) of Nduf, ribosomal protein (RP) and other mRNAs between WT and Larp1 cKO brains. Significance by t-test comparison between each class and Other mRNAs. **B**. Validation of changes in synaptosome enrichment in Larp1 cKO brains by qPCR. Levels of the indicated mRNAs in input and synaptosome samples from Larp1 WT and cKO brains were quantified by qPCR (n=6 for each genotype, error bars are SEM, significance by unpaired t-tests).
